# Effects of Biofeedback in Preventing Urinary Incontinence and Erectile Dysfunction after Radical Prostatectomy

**DOI:** 10.3389/fonc.2018.00020

**Published:** 2018-02-26

**Authors:** Fabiana S. B. Perez, Nathalia C. Rosa, Adson F. da Rocha, Luciana R. T. Peixoto, Cristiano J. Miosso

**Affiliations:** ^1^Medical Sciences Graduate Program, University of Brasilia, Brasilia, Brazil; ^2^Department of Physiotherapy, Alfredo Nasser College, Aparecida de Goiania, Brazil; ^3^Biomedical Engineering Graduate Program, University of Brasilia, Brasilia, Brazil

**Keywords:** radical prostatectomy, erectile dysfunction, urinary incontinence, biofeedback intervention, preoperative care

## Abstract

In this study, we present a biofeedback method for the strengthening of perineal muscles during the preoperative procedures for radical prostatectomy, and we evaluate this technique as a prevention measure against complications such as urinary incontinence (UI) and erectile dysfunction (ED), which affect prostatectomy patients after surgery. In the experimental protocol, the patients performed specific exercises with the help of a device that provided the patient with visual biofeedback, based on a plot of the anal pressure. For the experimental protocol, we selected 20 male patients, with an average age of 64.0 years, and submitted them to ten therapeutic sessions each. A control group consisting of 32 men with an average age of 66.3 years, who were treated with the same surgical procedure but not with the preoperative procedures, also took part in the experiment. To evaluate UI and ED after the surgery in both control and experimental groups, we used two validated questionnaires—to assess UI, we used the King’s Health Questionnaire (KHQ) and, for ED, we used the International Index of Erectile Function (IIEF-5) Questionnaire. We compared the variables associated with UI and ED after the surgery for the control and experimental groups. The occurrence of UI after radical prostatectomy in the control group (100% of the patients) was higher than that for the experimental group (5% of the patients), with *p* < 0.0001. Likewise, the occurrence of erectile dysfunction after prostatectomy in the control group (48.6% of the patients) was higher than that for the experimental group (5% of the patients), with *p* < 0.0001. The number of nocturia events also decreased as a consequence of the intervention (*p* < 0.0001), as did the number of disposable underwear units for urinary incontinence (*p* < 0.0001). Furthermore, we compared, only for the experimental group, the anal pressure before the biofeedback intervention and after the surgery, and we verified that the anal pressure after surgery was significantly higher (*p* < 0.0001). The results strongly suggest that the preoperative biofeedback procedure was effective in decreasing urinary incontinence and erectile dysfunction after radical prostatectomy. As future work, we intend to extend this analysis for larger samples and considering a broader age range.

## 1 Introduction

Prostate cancer affects around 13.5% of the male population over the age of 60 in the following 30 years, according to recent international polls (1). It usually results in need of chemotherapy and surgical partial or complete removal of the prostate (2), which in turn can lead to the loss of erectile function and urinary incontinence (3–6).

The prostate is an organ that is exclusive to the male gender, and which is located on the basis of the bladder and below the rectum, with the first region of the urethra traversing its volume. The prostate tissues include smooth muscles and fiber tissues, besides glands that produce part of the seminal fluid responsible for feeding the sperms (7, 8).

Prostatectomy consists of the removal of the entire prostate, the seminal vesicle, and a small part of the bladder. The surgery can cause resection of the internal sphincter as well as a lesion in part of the external sphincter. This injury frequently results in complications, such as the urinary incontinence (3, 9, 10) and the erectile dysfunction (3, 7, 8, 10–12).

The urinary incontinence (UI) resulting from prostatectomy can be temporary or persistent, which may depend on the level of the lesion affecting the distal sphincter in the surgical procedure (9, 13).

Prostatectomy can also result in damage to the cavernous arteries and nerves, thus leading to sexual dysfunction with loss of penile erection. According to Abdollah et al. (14), the erection dysfunction affects 95% of the operated men over 70 years of age, 50% of the patients aged between 55 and 65 years, and from 15 to 20% of patients aged less than 55 years (5).

In erectile dysfunction (ED), the vascular reflex mechanism is not able to pump blood with enough pressure toward the cavernous body of the penis, and consequently the penis erection cannot be maintained (5). Also, studies suggest that after prostatectomy the ischiocavernosus muscle, which is responsible for the rigidity phase of the erection, becomes weakened by the use of the bladder catheter, and that this weakening may also contribute to ED (4, 7, 8, 12).

It is important to emphasize that the occurrence of UI and ED may depend on several different factors, such as the perineal muscle tone preliminarily to the surgery, surgical intercurrences, patient age, and comorbidities. However, in our literature investigation, we did not find systematic studies evaluating the relation between preoperative therapies for perineal stimulation and the occurrence or not of postoperative UI or ED.

Therefore, in this research, we evaluate the effects of our proposed biofeedback therapy performed as a preoperative procedure, and assess its impact regarding UI and ED cases after prostatectomy. We start by describing the basis of biofeedback, our proposed methodology, including the proposed biofeedback protocols and session distributions, and our experimental evaluations. Next, we describe our measurements before and after the prostatectomy, regarding patients who participated in our research. Furthermore, we compare the outcomes of the surgeries associated to the proposed intervention with those patients in a control group, who were not treated with the biofeedback protocol.

## 2 Biofeedback for Pelvic Floor Stimulation

Biofeedback aims at providing consciousness about activity, and hence maximizing the muscle contractions in the pelvic floor region while avoiding other muscle groups’ contractions. In order to do so, we translate the intensity of muscle contractions into visual signals that are provided back to the subject performing the contractions. The biofeedback itself consists of a subject receiving this visual information about his own contractions, and then using such visuals to control the next contractions. It is a well-known fact that the use of visual biofeedback can improve a person’s ability to perform muscle activity, by visualizing the effects of his or her efforts and hence responding in a guided way in the following stages (15–17).

This is the reason why many urologists request physiotherapy urological sessions for perineal strengthening in prostate cancer patients before surgery. In fact, this process of bringing consciousness about pelvic muscles contractions improves strengthening of the specific desired muscles and helps reduce the UI and ED incidences (there is, however, a shortage of systematic research quantifying this aspect so far). We have also observed that, even when UI and ED occur, patients who took part in biofeedback perineal strengthening protocols experience more ease in solving these problems later. In this paper, we wish to evaluate the first of these aspects.

## 3 Methods

We selected 20 male volunteers with prostate cancer for whom was prescribed a biofeedback physiotherapeutic treatment before prostatectomy. A control group (*n* = 32) formed by men, with an average age of 66.3 years, who were treated with the same surgical procedure but not with the preoperative procedures also took part in the experiment. We compared the outcomes of their treatments with our intervention’s results.

Inclusion criteria for the experimental group were as follows: men with prostate cancer (with an encapsulated tumor), or with a history of this disease, diagnosed at an early stage; all participants must be in the preoperative procedure for radical prostatectomy with the same open surgical technique. For the control group, the participants went through the surgery, but without the preoperative biofeedback procedure.

In both groups, we excluded from the study patients with the following characteristics: metastasis; diabetes or decompensated hypertension; history of psychiatric diseases; will undergo a robotic or laparoscopic surgical procedure or a different procedure from the open surgery technique adopted; under treatment by radiotherapy or chemotherapy; and severe alcoholism.

All patients were submitted to open prostatectomy preserving the pudendal nerve, and not to the laparoscopic or robotic variety. A single surgeon has operated on all the patients in the experimental and the control groups, following the same procedures for both groups. The urological surgeon referred the patients to the CEREI physiotherapy clinic, where they went through our proposed protocol before surgery. Also, we evaluated the UI and ED after the surgical procedure in all the patients in the control and experimental groups.

The experimental protocol followed the current Brazilian legislation as well as the principles stated in the Declaration of Helsinki. The Research Ethics Committee of the Alfredo Nasser College approved the protocol (CAAE N. 61829516000008011). Each volunteer signed the free and informed consent statement, after being properly informed about the treatment protocol and the research objectives. At the first stage of the evaluation, each selected volunteer in the experimental group went through a urological assessment based on biofeedback.

**Figure 1:**
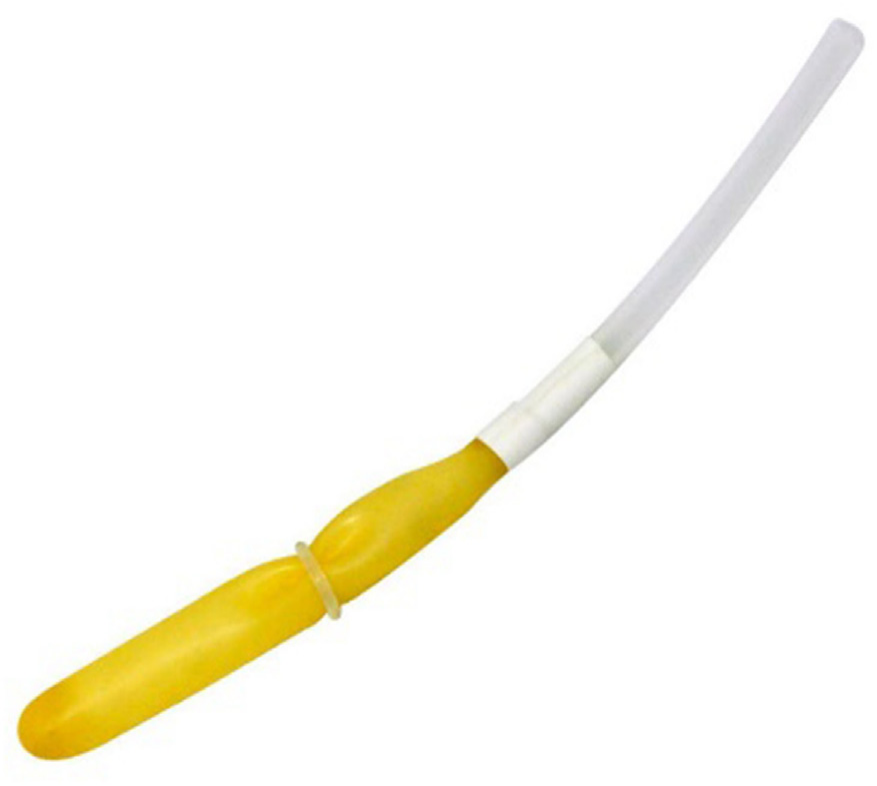
Inflatable anal latex probe used with the Neurodyn Evolution biofeedback device (Ibramed, Amparo, Sao Paulo, Brazil).

We then conducted ten physiotherapy sessions using the Neurodyn Evolution biofeedback device (Ibramed, Amparo, Sao Paulo, Brazil). The sessions occurred in ten consecutive working days. We also used an inflatable anal probe made out of latex (with register number 10360310013). This probe was surrounded by an initially non-lubricated condom, which was later lubricated using an appropriate contact gel (Carbogel). We introduced the probe into the anal canal and inflated it until the patient reported a mild discomfort and was able to feel the whole external probe surface. Figure 1 illustrates the probe used. Note that during the biofeedback session, we did not allow the volunteer to use accessory muscles, such as abductors, abdomen, and gluteus. After each physiotherapy session, we disposed of the condom and proceeded to the probe hygiene routine, according to the manufacturer instructions.

**Figure 2:**
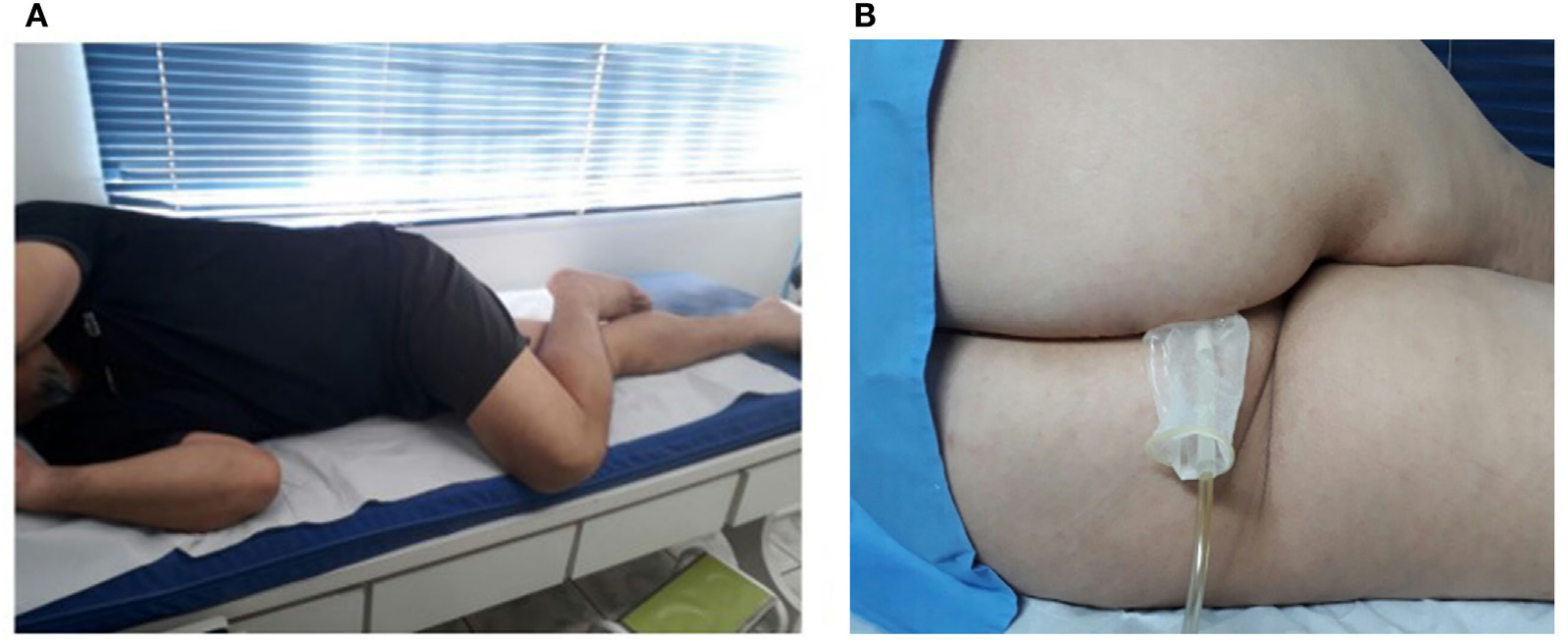
**(A)** Patient positioning (right lateral decubitus position) for both the taring and biofeedback stages. **(B)** Probe insertion using the decubitus positioning (here inverted for probe visualization).

During both the pressure taring and the two sets of voluntary contractions, described below, we requested the volunteer to get into the right lateral decubitus position, with one leg extended and the second flexed over the first one, as illustrated in Figure 2A. This positioning helps the opening of the anal canal so that the probe can be introduced into the rectum, as shown in Figure 2B. Also, the volunteer stayed at a distance of 3 m from a computer screen, so that he could always see the taring screen and the pressure reference signals, as described below.

**Figure 3:**
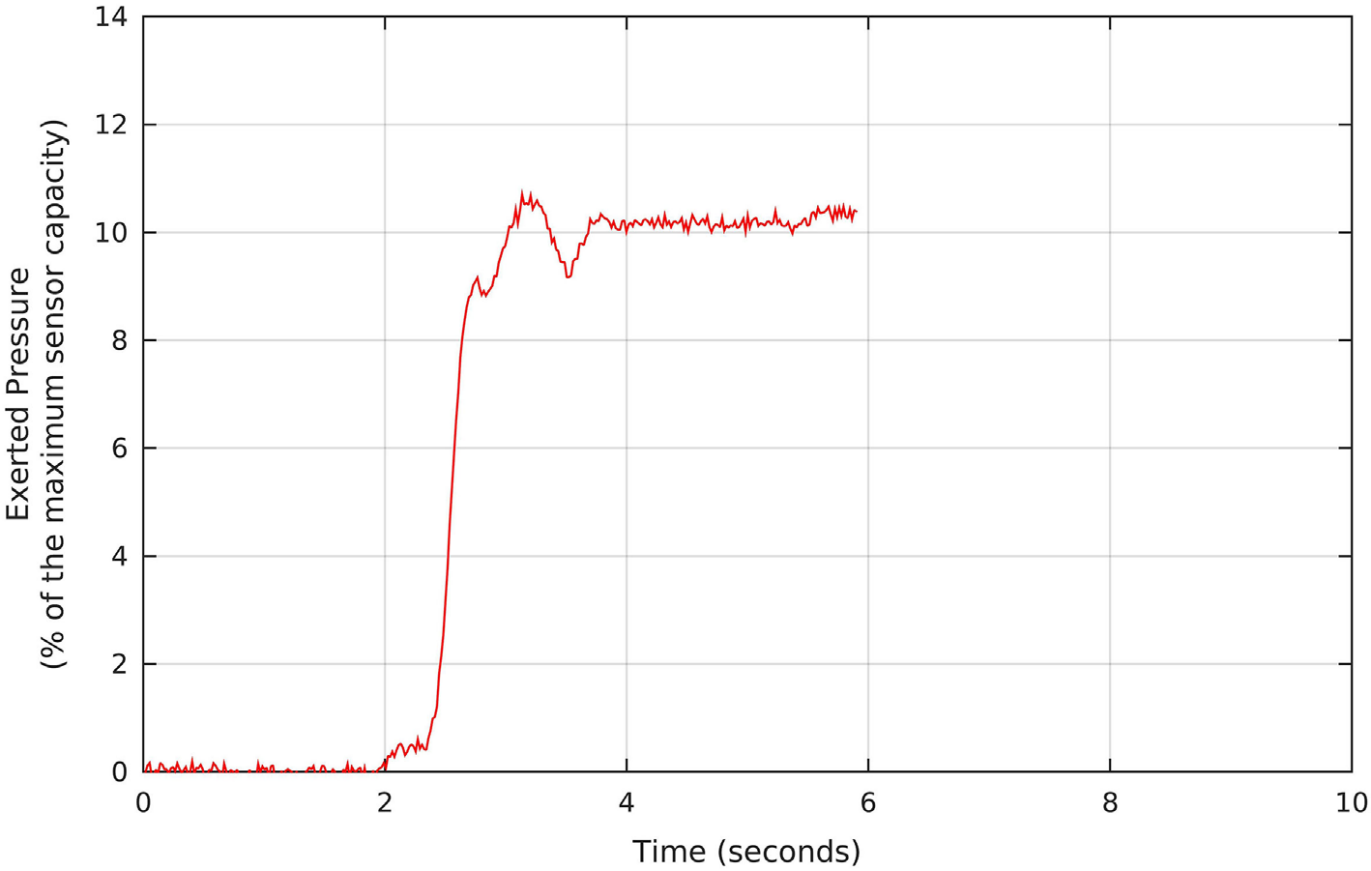
Example of pressure signal measured during the pressure taring, which precedes the biofeedback protocols. The Neurodyn Evolution screen shows the maximum voluntary contraction that each volunteer manages to apply to the anal probe (in this example, around 10% of the maximum sensor capacity). The procedure is repeated for a total of three times, and we take the maximum MVC, and use it as a reference for defining the target pressure during the biofeedback sessions.

The therapy started with pressure taring, as we requested each participant to perform three maximum voluntary contractions (MVCs). Figure 3 shows a screen of the Neurodyn software used to measure the anal pressure achieved. At the physiotherapist’s request, the patient reaches his MVC (in the example, 10% of the maximum sensor capacity), which is measured and stored for further analysis. In the process, we instructed the patient to try to reach the maximum contraction and to watch the pressure on the screen and, when the pressure starts to drop, to release the contraction and, right after the release, to repeat the contraction. Each participant performed three contractions, and we registered the maximum MVC value for each one.

**Figure 4:**
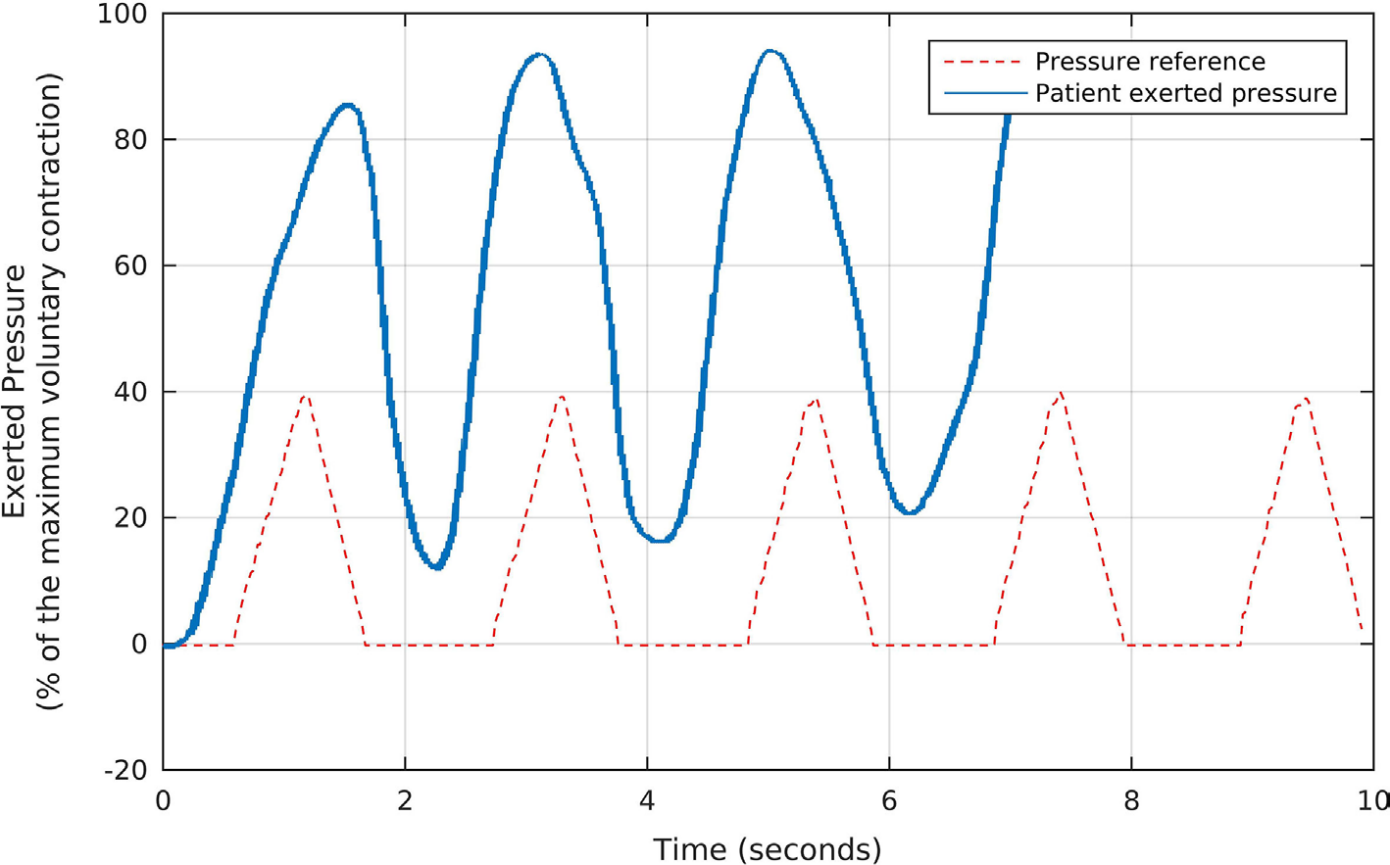
The fast, triangular-shaped pressure waveforms used as the reference for each volunteer during the first stage of the biofeedback sessions, and the corresponding pressures exerted by a patient. We oriented each participant to try and reproduce the reference waveforms by exerting pressure over the anal probe, while watching the generated signals in real time. Note that, as requested, the patient tried to maintain the actual pressure values above the minimum reference, while following the basic triangular shapes.

About 7 s after the taring session, we started the first part of the strengthening protocol, which consists of 7-min rapid contractions to stimulate type-II fibers. To conduct this procedure, we used a preprogrammed sequence of 5 triangular-shaped target pressure waveforms, which the Neurodyn software presented sequentially to the volunteer, as illustrated in Figure 4. Each triangular reference signal varied from 0 to 40% of the patients’ MVC. The patient was requested to try to reproduce the triangular shapes with the maximum possible offset, by exerting the appropriate pressures over the probe, according to the biofeedback principle of watching the results in real time and comparing them to the target pressures. Figure 4 shows, in blue, the actual pressures exerted by one of the volunteers during the first part of our protocol. Note that, as requested, the patient tried to maintain the actual pressure values above the minimum reference, while following the basic triangular shapes.

**Figure 5:**
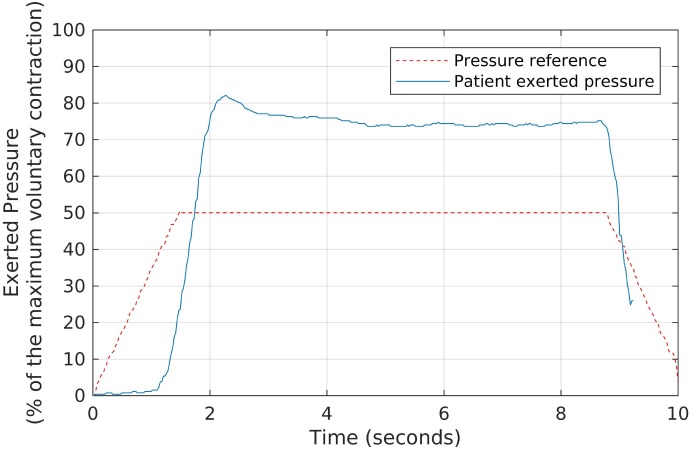
The slow, sustained pressure waveform used as the reference for each volunteer during the second stage of the biofeedback sessions, and the corresponding pressures exerted by a patient. We oriented each participant to try and keep a sustained pressure level above the reference waveform, by exerting pressure over the anal probe, while watching the generated signals in real time.

In the second part of the strengthening protocol, each patient performed, for a period of 6-min, a series of slow contractions, aimed at stimulating type-I fibers. In this case, the same Neurodyn software reproduced slower target shapes, with sustained pressure plateaus as shown in Figure 5. The plateaus were configured at 50% of the MVCs, and there was a 1-min interval between contractions to avoid muscle fatigue. Each patient was requested to try and keep a pressure plateau above the minimum of 50% of the MVC.

At each physiotherapy session, we stored the maximum attained pressures in a report; so that after the 10 sessions, we provided this information both to the volunteer and to his surgeon, who could verify the evolution of the perineal muscles with the proposed protocol. The prostatectomy happened within a few days after the tenth physiotherapy session.

For conducting the UI and ED evaluations after prostatectomy in the control and experimental groups, we used two specific, validated questionnaires. In the case of UI, we used the King’s Health Questionnaire (KHQ), which is validated for use both with male and female patients (18, 19). It includes all the questions belonging to the Overactive Bladder Syndrome Score (OABSS), as well as additional questions. Also, it computes scores that are divided into eight main domains, and higher scores in each domain indicate worse life conditions due to UI. We emphasize that the International Continence Society (ICS) considers the KHQ highly recommended for clinical research (20).

In evaluating ED, on the other hand, we used the International Index of Erectile Function (IIEF-5) questionnaire (21), which is a well-known, validated instrument for evaluating ED. In this case, each patient receives a final score ranging from 5 to 25, with lower values indicating severe ED and higher scores indicating low or absent ED.

In addition, during the urological assessment by the physiotherapist who conducted the physiotherapy sessions, additional data were collected using a supplemental questionnaire (a standard urological form developed at the Federal University of Sao Paulo, Brazil) and the anal pressure measurements.

For both control and experimental groups, we obtained, after the prostatectomy, information on the average daily number of nocturia events (the average number of times the patient wakes up to urinate), and the daily number of protectors used by each patient (the protector was a disposable underwear for male urinary incontinence). Also, for both groups, the patients were classified, after the surgery, as having or not having UI and/or ED. Similar data were also collected for the control group and also presented in a table. Tables 1–4 present the data for both groups.

**Table 1:** Control group’s measured data regarding age, postoperative anal pressure, urinary incontinence occurrence, number of nocturia events, erectile dysfunction occurrence, and daily number of protectors (disposable underwear for male urinary incontinence) used after prostatectomy.

Volunteer	Age (years)	Postoperative anal pressure (mmHg)	Postoperative UI occurrence	Number of nocturia events	Postoperative ED occurrence	Daily number of protectors
C1	68	22	1	2	0	3
C2	74	3	1	3	0	6
C3	72	26	1	1	1	3
C4	66	21	1	1	1	12
C5	67	21	1	3	1	4
C6	53	10	1	3	0	5
C7	63	26	1	3	0	3
C8	76	11	1	2	1	7
C9	65	3	1	2	0	3
C10	71	39	1	2	1	0
C11	70	4	1	7	0	1
C12	48	14	1	4	1	2
C13	68	17	1	2	0	3
C14	67	9	1	3	1	3
C15	71	21	1	2	1	2
C16	66	18	1	2	1	2
C17	63	10	1	3	1	4
C18	70	21	1	1	1	3
C19	65	21	1	2	0	4
C20	67	5	1	3	0	5
C21	66	11	1	2	0	7
C22	62	15	1	1	0	3
C23	68	24	1	2	1	2
C24	69	27	1	0	1	4
C25	67	32	1	1	1	3
C26	74	10	1	2	0	6
C27	57	4	1	0	0	8
C28	70	14	1	4	0	6
C29	61	14	1	0	1	2
C30	65	9	1	3	1	2
C31	63	16	1	2	1	5
C32	69	75	1	3	0	2
Average	66.3	17.9	1.0	2.2	0.53	3.9
SD	5.8	13.6	0	1.4	0.5	2.4

*In the columns that show UI and ED occurrence, 1 (one) indicates occurrence, and 0 (zero), non-occurrence*.

For the experimental group, we measured the maximum anal pressure at two different instants. The first measurement was taken at the beginning of the first physiotherapy session and, the second, after the prostatectomy (more precisely, after the removal of the urethral probe). The results of these measurements are shown in Table 2.

**Table 2:** Experimental group’s measured data regarding age, anal pressures before and after prostatectomy, urinary incontinence (UI) occurrence after prostatectomy, number of nocturia events after prostatectomy, erectile dysfunction (ED) occurrence after prostatectomy, and daily number of protectors used by each patient (disposable underwear for male urinary incontinence).

Volunteer	Age (years)	Preoperative anal pressure (mmHg)	Postoperative anal pressure (mmHg)	Postoperative UI occurrence	Number of nocturia events	Postoperative ED occurrence	Daily number of protectors
E1	61	13	41	0	0	0	0
E2	65	24	47	0	0	0	0
E3	69	4	43	1	1	0	2
E4	54	10	51	0	4	0	0
E5	63	27	49	1	0	1	0
E6	60	12	98	0	1	0	0
E7	68	23	92	0	0	0	0
E8	64	29	67	0	0	0	0
E9	65	4	70	0	0	0	0
E10	69	6	42	0	3	0	0
E11	70	9	47	0	0	0	0
E12	64	9	44	0	0	0	0
E13	65	15	66	0	3	0	0
E14	62	10	43	0	0	0	0
E15	73	25	43	0	1	0	0
E16	55	16	41	0	0	0	0
E17	65	3	21	0	0	0	0
E18	63	5	22	0	0	0	0
E19	63	28	43	0	0	0	0
E20	62	30	77	0	0	0	0
Average	64.0	15.1	52.4	0.10	0.7	0.05	0.1
SD	4.6	9.4	20.2	0.3	1.2	0.2	0.4

*In the columns that show UI and ED occurrence, 1 (one) indicates occurrence, and 0 (zero), non-occurrence. The preoperative anal pressure was measured before the ten physiotherapy sessions*.

Hypothesis tests were performed to assess the statistical significance of results found with the tests that were used to quantify the improvement due to the proposed biofeedback intervention. When we saw improvements (reduction) in the average number of nocturia events and of protectors used by each patient, we tested for statistical significance of these improvements. In the comparisons, we used the Lilliefors normality test (LT) to evaluate the null hypothesis that these variables had a normal distribution. Since the normality was rejected (both in the control and experimental groups and for all tested variables), we then used a non-parametric test—the unpaired Wilcoxon Rank Sum test—to compare the UI and the ED in the control and experimental groups. We rejected the null hypotheses whenever *p* < 0.05.

We also assessed the effect of the biofeedback intervention on the occurrence of UI and ED after the surgery, and we evaluated the statistical significance of these improvements. The variables that indicate occurrence of UI and ED are binary, where 1 (one) indicates occurrence and 0 (zero), non-occurrence. Because of this binary nature of these variables and because the experimental sample is small, we used the Fisher’s exact test to test for statistical significance. We rejected the null hypothesis whenever *p* < 0.05.

For the experimental group, we compared the anal pressures measured before the biofeedback intervention and after the prostatectomy. To do so, we first tested for normality in the data distribution, by using the Lilliefors test and, since the normality was rejected, we used the paired Rank Sum Wilcoxon test. The null hypothesis was rejected whenever *p* < 0.05.

We used MatLab^®^ for all the statistical tests in this research.

## 4 Results and Discussion

The selected volunteers in the experimental and control groups were, on average, 64.0 and 66.3 years old, respectively.

Regarding the control group, Table 1 summarizes the measured data, including the postoperative anal pressure, the UI occurrence variable, the average daily number of nocturia events (the average number of times the patient wakes up to urinate), the ED occurrence variable, and the daily number of protectors used by each patient (the protector was a disposable underwear for male urinary incontinence). Note that here we define the UI occurrence variable as 1 (one) if the patient reported UI, and 0 (zero) otherwise; similarly, we set the ED occurrence value as 1 (one) if the patient reported ED, and 0 (zero) otherwise. These occurrences were collected during the urological assessment by the physiotherapist who conducted the physiotherapy sessions, using a questionnaire and the measurements of the anal pressures.

Concerning the experimental group, our preliminary evaluation indicated no perineal dysfunction, and the patients started the preoperative preparations within a week after the prostate cancer was confirmed by Prostate-Specific Antigen (PSA) exams, rectal touch, pelvic ultrasonography, and prostate biopsy. All the volunteers in this group took part in the proposed anal biofeedback physiotherapy, with a total of 10 sessions.

Table 2 shows the results for all 20 patients in the experimental group. The table presents data regarding age, anal pressures before the biofeedback intervention and after prostatectomy, urinary incontinence (UI) occurrence after prostatectomy, number of nocturia events after prostatectomy, erectile dysfunction (ED) occurrence after prostatectomy, and daily number of protectors used by each patient (disposable underwear for male urinary incontinence). In the columns that show UI and ED occurrence, 1 (one) indicates occurrence and 0 (zero), non-occurrence. The preoperative anal pressure was measured before the ten physiotherapy sessions.

The assessment of the outcomes of the biofeedback intervention was also made using the KHQ and the IIEF-5 questionnaires. Table 3 presents a statistical summary of the results of the KHQ applied to the control and the experimental groups. The average values and the SDs of each different area of the questionnaire are presented. The raw data in this questionnaire were used to assess the occurrence of UI in both groups.

**Table 3:** Statistical summary of the results of the KHQ applied to the control group (CG) and experimental group (EG), in terms of the scores related to the different considered areas.

Patient	General health perceptions	Incontinence impact	Role limitations	Physical limitations	Social limitations	Personal relationships	Emotions	Sleep/energy	Severity measures
**CG**									
Av.	72.7	84.4	76.0	91.1	61.5	59.8	67.7	83.3	77.1
SD	19.4	23.9	17.9	11.2	14.7	19.6	15.7	18.9	7.6
**EG**									
Av.	61.2	58.3	56.7	74.2	45.0	60.0	35.0	14.2	44.2
SD	17.2	23.9	20.5	20.6	12.2	19.1	22.0	15.5	13.3

*The table shows the average values (Av.) and SD of each category*.

Table 4 presents a statistical summary of the results of the IIEF-5 questionnaire applied to the control and experimental groups. The raw data in this questionnaire have been used to assess the occurrence of ED in both groups. The table presents, for both control and experimental groups, the IIEF-5 scores over the five considered ranges. Based on Table 4, we were able to perform a more detailed evaluation of the outcomes for the groups, in terms of ED. We note that the IIEF-5 scores in the experimental group are higher than those in the control group. In fact, 90% of the scores in the experimental group are in the 22–25 range, whereas in the control group only 45.7% of the scores are in this range, the others being distributed mainly over the 5–7, 8–11, and 12–16 ranges.

**Table 4:** Distribution of the IIEF-5 scores over the 5 considered ranges, for both the control and the experimental groups.

IIEF-5 Score range	Percentage of volunteers in the control group	Percentage of volunteers in the experimental group
5–7	8.6	5.0
8–11	11.4	0.0
12–16	11.4	0.0
17–21	5.7	5.0
22–25	45.7	90.0

We observe, in Table 2, an apparent increase of anal pressure for all patients, later confirmed by a hypothesis test as described below. Also, there were just two cases of UI and only one case of reported ED after surgery. It is important to observe that these values are lower than what we commonly see in our clinical practice when no biofeedback treatment is provided during the preoperative stages.

We compared the results in Table 2 (for the experimental group) to those of Table 1 (for the control group) using the procedure for hypothesis testing described in the Methods section.

In two of the comparisons between data from the experimental and control groups, our null hypotheses were that (i) the occurrence of UI after radical prostatectomy in the control group is equal to that in the experimental group; (ii) the occurrence of ED after radical prostatectomy in the control group is equal to that in the experimental group. Table 5 shows the p-values obtained. Since both UI and ED occurrences markedly decreased as a result of the intervention, and since both p-values were very small, these results suggest that the preoperative biofeedback intervention significantly reduced the occurrences of both erectile dysfunction and urinary incontinence after radical prostatectomy, for the tested modality (open surgery preserving the pudendal nerve).

**Table 5:** *p*-values obtained regarding the comparisons between the experimental group (submitted to the proposed preoperative biofeedback intervention) and the control group.

Null hypothesis	*p*-Value
The median of the occurrence of urinary incontinence after radical prostatectomy in the control group is equal to that in the experimental group	4.5 × 10^−12^
The median of the occurrence of erectile dysfunction after radical prostatectomy in the control group is equal to that in the experimental group	3.1 × 10^−4^
The median of the number of nocturia events after radical prostatectomy in the control group is equal to that in the experimental group	8.2 × 10^−5^
The median of the number of protectors used after radical prostatectomy in the control group is equal to that in the experimental group	3.1 × 10^−9^

Table 2 also shows the number of nocturia events (the number of times that the patient wakes up at night to urinate). Only six patients had at least one event, and the average number of events among the 20 patients was only 0.65 event per night. On the other hand, the number of events in the control group, shown in Table 1, had an average of 2.22 events per night, and 29 out of 32 patients had at least one event per night. This result suggests a clear decrease in the number of events as a consequence of the feedback preoperative intervention. In fact, the number of nocturia events per night is usually higher than one per night, after prostatectomy, and most patients report UI and/or ED after prostatectomy when no biofeedback physiotherapy is conducted during the preoperative preparations (3, 9, 11, 14, 22–24). To test the statistical significance of this result, we followed the procedure described in the Methods section, which resulted in p = 8.2 × 10^−5^. This result is also presented in Table 5. Therefore, the biofeedback intervention resulted in a statistically significant reduction in the average number of nocturia events.

The last columns of Tables 1 and 2 show the daily number of protectors used, respectively, by the control and by the experimental group. For the control group, 31 out of 32 participants used protectors, and the daily average number of protectors used was 3.91 protectors per day. This high value is associated with the fact that all 32 subjects had incontinence after surgery. For the experimental group, the only volunteer that used protectors was the only one in the experimental group that was considered incontinent, and the daily average number of protectors within the experimental group was 0.10 protectors per day. Thus, there is a clear difference regarding the number of protectors used, and we performed the hypothesis test described in the Methods section. The test showed that the mean number of protectors used in the experimental group is significantly lower than the number used in the control group, since *p* = 3.1 × 10^−9^ (the result is also shown in Table 5). This is an additional evidence for the effectiveness of the intervention proposed.

Besides the tests that compared the outcomes of the intervention proposed with the outcomes of a surgery in which there was no intervention, we also analyzed the experimental group outcomes in terms of possible increases in anal pressure between the beginning and the end of the intervention. The results in Table 2 suggest a clear increase of anal pressure after the physiotherapy sessions and the prostatectomy—the average value of the anal pressure, in mmHg, before the physiotherapy sessions was 15.1, and the mean after the surgery was 52.4. To check for statistical significance, we applied the procedure described in the Methods section to test the following null hypothesis: the median of the anal pressures before the beginning of the physiotherapy sessions was equal to that after the surgical procedure. The test resulted in *p* = 5.1 × 10^−7^ (the result is also shown in Table 6). These results reinforce the evidence that there was an important improvement over patients not submitted to the biofeedback protocol before surgery.

**Table 6:** *p*-Value obtained for the hypothesis regarding increase in anal pressure for patients submitted to the proposed protocol.

Null hypothesis	*p*-Value
The median of the anal pressures at the end of the sessions was equal to that at the beginning	5.1 × 10^–7^

## 5 Conclusion

Our results and statistical analyses suggest the physiotherapeutic intervention using our biofeedback protocol helps preventing perineal dysfunctions which are common after prostatectomies, such as urinary incontinence and erectile dysfunctions. Among all the research volunteers, only two presented urinary incontinence after the removal of the urethral probe, and just one presented erectile dysfunction. Regarding enureses occurrences, only three reported waking up three or four times at night to urinate, whereas all other participants showed less than three cases.

The patients diagnosed with prostate cancer may be submitted to surgery for a total prostate removal, and this process may be preceded by a physiotherapy intervention. By using biofeedback, this therapy may generate an awareness of the perineal muscles, the ones that are weakened after the surgery. Biofeedback may also cause a hypertrophy of the sphincter that surrounds the male urethra, around the prostate to be removed. This preliminary hypertrophy assists the surgeon regarding avoiding causing further damage to this muscle when extirpating the prostate. We also observe, in our clinical experience, that patients who prepare the perineal muscles before surgery have improved postoperative results concerning common complications (UI and ED), which otherwise cause severe impairments.

## Ethics Statement

Ethics Commitee that approved our study: Research Ethics Committee of the Alfredo Nasser College. Number of the approval document provided by the Ethics Committee: CAAE N. 61829516000008011. All human participants signed a free and informed consent letter, after being informed of all the procedures they would undertake, and before taking part in the experiment.

## Author Contributions

FP participated in the experimental design and performed the main experimental procedures, including all the patient interventions. She also took part in the discussion of the results and wrote parts of the manuscript. NR participated in the experimental procedures (data acquisition), discussion of the results, and preparation of parts of the manuscript. AR, LP, and CM participated in the experimental design and the statistical analysis and wrote sections of the manuscript. They also participated in the discussion of the collected data and statistical analysis. CM was the principal advisor of this research. All authors contributed to the manuscript and read and approved the full text.

## Conflict of Interest Statement

The authors declare that the research was conducted in the absence of any commercial or financial relationships that could be construed as a potential conflict of interest.
